# Food-Grade Liposome-Loaded Delivery Systems: Current Trends and Future Perspectives

**DOI:** 10.3390/foods14172978

**Published:** 2025-08-26

**Authors:** Erkan Mankan, Canan Yagmur Karakas, Oznur Saroglu, Mondher Mzoughi, Osman Sagdic, Ayse Karadag

**Affiliations:** 1 Food Engineering Department, Chemical, and Metallurgical Engineering Faculty, Yildiz Technical University, 34210 Istanbul, Türkiye; erkan.mankan@trustlifeventures.com (E.M.); yagmur.karakas@yildiz.edu.tr (C.Y.K.); osaroglu@yildiz.edu.tr (O.S.); osagdic@yildiz.edu.tr (O.S.); 2 Trustlife Labs Drug Research & Development Center, 34774 Istanbul, Türkiye; mzoughi17@itu.edu.tr

**Keywords:** encapsulation, food-grade ingredients, nutraceuticals, health-oriented food

## Abstract

Liposomes have emerged as versatile carriers in the food industry due to their amphiphilic structure, biocompatibility, and ability to encapsulate both hydrophilic and lipophilic bioactive compounds. They offer promising benefits by enhancing the solubility and bioavailability of food ingredients such as vitamins, polyphenols, carotenoids, peptides, and omega-3 fatty acids. However, liposomes in aqueous form often suffer from poor stability, leakage of encapsulated compounds, and sensitivity to environmental conditions. To address these challenges, hybrid delivery systems have been developed by incorporating liposomes into various solid or semi-solid encapsulation matrices such as nanofibers, particles, cast films, hydrogels, and emulsions. These combinations can offer synergistic advantages, including improved structural integrity, enhanced protection during processing and storage, extended-release profiles under digestive conditions, and versatile applicability across different applications. This review comprehensively discusses liposome structure, preparation methods, and their incorporation into various encapsulation matrices, focusing exclusively on food-grade ingredients. It highlights recent advancements in hybrid liposome-based systems tailored for food applications, with an emphasis on their functional performance and delivery efficiency. Overall, these hybrid systems hold great promise for developing next-generation functional foods with improved health benefits and shelf stability.

## 1. Introduction

Liposome technology is a versatile platform for delivering bioactive compounds in pharmaceutical and food applications. Liposomes are spherical vesicles made up of one or more phospholipid bilayers, capable of encapsulating hydrophilic, hydrophobic, and amphiphilic substances. Specifically, hydrophilic molecules are entrapped within the aqueous core, while lipophilic molecules are embedded in the bilayer. Their structural similarity to biological membranes makes them biocompatible and generally safe for ingestion [1]. According to the ISO/TS 80004-1:2015 standard and the European Commission Recommendation 2011/696/EU, nanoscale materials have at least one external dimension between 1 and 100 nm [2,3]. While many small unilamellar vesicles (SUVs) fall within this range, larger vesicles such as large unilamellar vesicles (LUVs) and multilamellar vesicles (MLVs) often exceed it; however, in food science literature, vesicles below approximately 500 nm are still commonly termed as “nanoliposomes” due to their improved colloidal stability and bioavailability [4].

Originally developed as drug carriers in the 1960s, liposomes have since been adapted for encapsulating nutrients and functional food ingredients to improve their stability and bioavailability. Encapsulation within liposomal vesicles can protect labile vitamins, antioxidants, enzymes, or flavors from degradation caused by exposure to light, oxygen, pH, or heat during food processing and storage [5]. Moreover, liposomes can be engineered for targeted delivery by modifying their surface with ligands or by tuning their size and charge. In this way, they can interact selectively with certain cells or tissues, thereby delivering nutrients or bioactives to specific sites [6]. This targeting ability, which is well demonstrated in pharmaceutical studies, is now being explored for functional foods to enhance the uptake of vitamins or antioxidants in the gastrointestinal tract. By adjusting the lipid composition or adding responsive elements, formulators can design liposomes that release their payload gradually or under specific conditions, thereby maintaining prolonged nutritional or preservative effects while minimizing burst release that could otherwise lead to losses or side effects [7].

However, the performance of liposomes depends heavily on their physicochemical stability. Although they must remain intact and well dispersed in the food matrix, liposomes in aqueous dispersions conventionally suffer from aggregation, degradation, hydrolysis, and oxidation reactions, which can cause the leakage of encapsulated compounds [8]. Extreme pH or high temperatures can disrupt the phospholipid bilayer, causing vesicle aggregation or rupture. To address these challenges, strategies such as coating liposomes with edible biopolymers (e.g., chitosan or alginate) to create an additional diffusion barrier [9,10] have been suggested. Converting liposomal preparations into more stable powdered forms using drying techniques like spray-drying or freeze-drying in the presence of stabilizing excipients (e.g., trehalose, sucrose, or biopolymers) to maintain vesicle integrity during dehydration and rehydration [5,11,12,13] is another approach to meet the challenges related to vesicle fusion, leakage, and loss of functional activity. Another promising approach involves integrating bioactive-loaded liposomes into other delivery systems, thereby preserving their integrity while leveraging the advantages of each method to mitigate individual limitations [14,15,16,17,18]. Although numerous reviews have addressed the physicochemical properties and functional potential of liposomes, including surface modifications, a comprehensive overview focusing specifically on their integration into hybrid delivery systems tailored for the food industry remains lacking. This review aims to fill that gap by providing a perspective on liposome structure, and preparation techniques, with the main focus on their incorporation into various encapsulation matrices developed exclusively from food-grade ingredients and key bioactives such as vitamins, polyphenols, carotenoids, peptides, and omega-3 fatty acids. Particular emphasis is given to the emerging trend of embedding liposomes within semi-solid and solid matrices, including hydrogels, electrospun nanofibers, cast films, particles, and emulsions, as a strategy to overcome the inherent limitations of conventional liposomes. Furthermore, this review highlights current applications of these liposome-loaded hybrid systems and underscores their multifaceted potential in the development of next-generation functional foods with enhanced health benefits and improved shelf-life stability.

## 2. Liposome Structure and Composition

**Figure 1:**
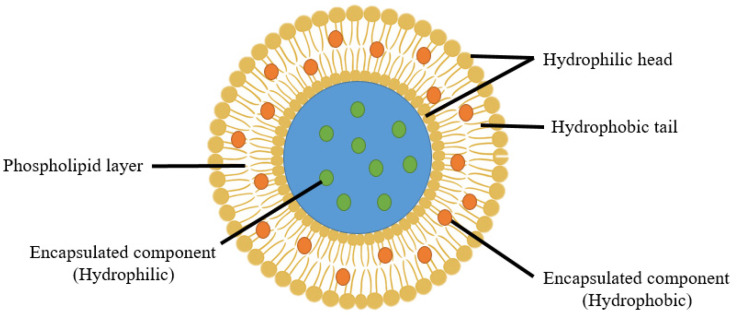
Structure of a liposome illustrating the phospholipid bilayer with hydrophilic heads, hydrophobic tails, and the localization of encapsulated hydrophilic and hydrophobic compounds.

Liposomes are spherical vesicles composed of one or more phospholipid bilayers enclosing an aqueous core (Figure 1). In these structures, the amphiphilic phospholipids orient with their hydrophilic heads facing outward the aqueous environment and their hydrophobic tails inward, enabling bilayer formation [19]. Liposome sizes typically range from 20 nm to several micrometers, most commonly between 50 and 500 nm. Key structural parameters such as lamellarity, surface charge, and lipid composition significantly influence the encapsulation efficiency, stability, and biological performance [20].

Liposomes can be formed from both natural and synthetic phospholipids, with lipid composition playing a crucial role in defining their physicochemical properties, including particle size, membrane rigidity, fluidity, and surface charge. For example, unsaturated phospholipids, like those derived from egg or soybean phosphatidylcholine, produce more fluid and permeable membranes. In contrast, saturated phospholipids like dipalmitoylphosphatidylcholine (DPPC) yield more rigid and less permeable bilayers [21]. The interaction between encapsulated bioactives and the lipid bilayer is another key factor influencing liposomal performance. Highly lipophilic compounds, such as curcumin, insert into the hydrophobic region of the phospholipid bilayer, enhancing membrane rigidity, reducing permeability, and modulating release kinetics, ultimately improving stability and bioavailability [22]. Conversely, hydrophilic compounds such as aqueous herbal extracts, peptides, or vitamins B and C are typically entrapped within the aqueous core, where they are shielded from degradation and released in a control manner. This polarity-based compartmentalization allows the co-delivery of multiple bioactives with different solubility profiles in a single delivery system [23]. Additionally, the phospholipid head group may carry positive, negative, or zwitterionic charges, contributing to colloidal stability through electrostatic repulsion. Variation in hydrophobic tail length, saturation, and symmetry further modulates the structural and functional properties of liposomal membranes [20,21]. Beyond their structural and functional properties, safety and regulatory considerations are crucial, particularly for application in food matrices. Many phospholipids used in liposome production (e.g., lecithin) are classified as Generally Recognized As Safe (GRAS) by the FDA and are widely used in the food industry [24,25]. However, nanosizing and the incorporation of other materials (e.g., surfactants, polymers) may alter their toxicological profiles. Therefore, comprehensive safety evaluations, including in vitro and in vivo toxicity studies, are needs to be considered [26].

**Figure 2:**
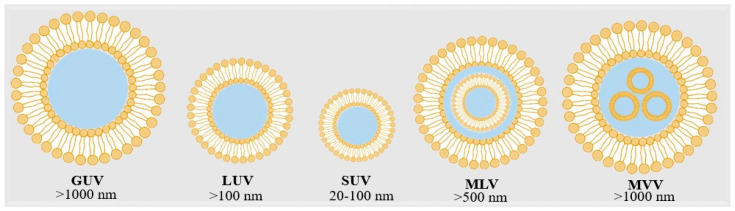
Types of liposomes based on lamellarity: small unilamellar vesicle (SUV), large unilamellar vesicle (LUV), multilamellar vesicle (MLV), multivesicular vesicle (MVV), and giant unilamellar vesicle (GUV).

Unilamellar vesicles (ULVs) possess a single phospholipid bilayer and are classified by size into small unilamellar vesicles (SUVs, 20–100 nm), large unilamellar vesicles (LUVs, 200–500 nm), and giant unilamellar vesicles (GUVs, >1000 nm) (Figure 2). SUVs are preferred for targeted delivery due to their small size and tissue penetration ability, while LUVs and GUVs are better suited for applications requiring prolonged circulation and sustained release [27,28]. Multilamellar vesicles (MLVs), comprising multiple concentric bilayers, offer higher encapsulation efficiencies, particularly for hydrophobic compounds, due to their greater phospholipid content and surface area. This multilayered structure also enhances stability and supports gradual release kinetics [29]. Multivesicular vesicles (MVVs) contain several discrete vesicles enclosed within a larger liposomal core, enabling simultaneous encapsulation and release of multiple agents, which is advantageous for complex therapeutic strategies [29].

Understanding these structural characteristics and compositional variations is essential for tailoring their functionality to specific applications. The interplay between vesicle size, lamellarity, lipid type, and surface properties directly influences encapsulation capacity, release kinetics, and biological stability [27]. These physicochemical attributes also govern the interaction between liposomes, their encapsulated compounds, and the surrounding medium [6]. Given these factors, selecting an appropriate production method is critical for achieving the desired liposomal characteristics. The following section therefore outlines various liposome fabrication techniques, emphasizing how processing parameters influence vesicle size distribution, lamellarity, and encapsulation efficiency in both laboratory-scale and industrial applications.

## 3. Liposome Preparation Methods

Liposome preparation techniques can be broadly categorized into conventional and novel approaches (Table 1). Conventional methods include widely established techniques such as thin-film hydration, reverse-phase evaporation, and ethanol injection. In recent years, novel approaches such as supercritical fluid-based techniques and microfluidics have also been applied to liposome production [30].

### 3.1. Conventional Methods

Thin-film hydration, also known as the Bangham method, is one of the most widely used small-scale techniques for liposome production. In this process, lipids and lipophilic active compounds are dissolved in volatile organic solvents (e.g., chloroform, methanol). Following solvent evaporation, a thin phospholipid film forms on the container wall. Subsequent hydration with an aqueous medium initiates the self-assembly of multilamellar vesicles, which often require a further size reduction process, such as sonication, extrusion, or high pressure homogenization, to achieve liposomes with uniform characteristics [31].

Reverse-phase evaporation is a modification of the thin-film hydration method. Lipids are initially dissolved in a suitable organic solvent and blended with an aqueous phase. The mixture is maintained above the lipid phase transition temperature while the solvent is gradually removed by evaporation. The resulting liposomal suspension is then homogenized to reduce particle size and improve vesicle uniformity [32].

Ethanol injection is commonly used for the production of small unilamellar vesicles. In this method, lipids are dissolved in ethanol and rapidly injected into a large volume of aqueous phase under vigorous stirring. The rapid dilution of a lipid–ethanol solution in water induces lipid reorganization at the ethanol–water interface, initially forming bilayer fragments that subsequently aggregate into vesicles. Parameters such as injection velocity, stirring rate, lipid concentration, and the ethanol-to-water ratio strongly influence encapsulation efficiency [28].

While effective, conventional methods have several limitations, including labor-intensive protocols, high-energy size-reduction steps, and exposure to organic solvents, all of which may compromise the stability or activity of both encapsulated compounds and lipids [20]. In pharmaceutical and food applications, the complete solvent removal of organic solvents is mandatory to meet safety and regulatory requirements, often necessitating additional purification and waste management steps, ultimately increasing production costs and complicating large-scale manufacturing [28].

**Table 1:** Comparison of common liposome preparation methods in terms of process characteristics, advantages, disadvantages, and vesicle types.

Method	Brief Description	Advantages	Disadvantages	Vesicle Types	Ref.
Thin-film hydration	Thin lipid film is hydrated to form multilamellar vesicles	Compatible with various lipid types; allows high lipid loading; well established	Low encapsulation efficiency for hydrophilic drugs; large vesicles; solvent removal	MLV, MVV	[31]
Reverse-phase evaporation	Solvent-dissolved lipids mixed with aqueous phase are homogenized after solvent removal	Ease of application; adequate encapsulation efficiency	High solvent use; time-consuming	MLV, LUV	[28]
Ethanol injection	Ethanol-dissolved lipids are injected into aqueous phase, where vesicles form upon dilution	Simple operation; high scalability; cost-efficient	Low lipid loading; hard to load macro molecules; solvent removal	SUV, MLV	[31]
Supercritical fluid	Liposomes are formed using supercritical CO_2_, which enables lipid processing through solvent-free or low-solvent conditions	Enables solvent-free encapsulation of sensitive compounds with controlled nanoscale release	High cost; precise control; poor hydrophilic drug loading	MLV	[33,34]
Microfluidics	Intense fluid mixing in micro- or nanoscale channels enables the formation of uniformly sized liposomes with a narrow particle size distribution	Ease of scaling up; targeted delivery; improved stability; enhanced bioavailability due to uniform nanosized liposomes	High equipment cost; viscosity sensitive; thermal degradation risk	SUV	[33]

### 3.2. Novel Methods

Emerging technologies such as supercritical fluid processing and microfluidics offer advanced control over liposome characteristics and scalability.

Supercritical fluid-based techniques utilize carbon dioxide (CO_2_) in its supercritical state to dissolve lipids, thereby eliminating the need for conventional organic solvents [20]. These approaches enable rapid, one-step preparation process with precise tuning of physicochemical properties. One such approach involves generating water droplets by spray atomization, which are subsequently coated with phospholipids. As these droplets descend into a water reservoir at the base of the vessel, liposome assembly is completed. This process emulsifies lipids in deionized water, followed by pressurization with supercritical fluid [33]. Supercritical fluids can serve multiple functions: acting as solvents or co-solvents for lipids, functioning as anti-solvents to trigger liposome formation upon hydration, and serving as dispersing agents to ensure uniform lipid distribution in the aqueous phase [34].

Microfluidic methods have more recently emerged as precise and scalable alternatives for liposome production [20,30]. This approach employs microscale channels to precisely control fluid flow. Lipids dissolved in ethanol or isopropanol are injected into the aqueous phase, either co-current or counter-current to the flow direction. Microfluidization offers several benefits, including low polydispersity, continuous solvent flow without clogging, compatibility with heat-sensitive compounds, and scalability for larger production volumes. Frequently, microfluidization is also applied as a post-processing step after conventional methods to reduce particle size and improve uniformity. Such processing can modify vesicle size and internal structure while simultaneously enhancing encapsulation efficiency, drug loading capacity, stability, and bioavailability [35]. The final particle size is influenced by factors such as operating pressure, processing temperature, and the number of passes through the microfluidizer [36]. On the other hand, excessive mechanical stress—arising from high pressure or repeated cycles—can damage the integrity of liposomes, leading to aggregation or the formation of multivesicular structures with larger sizes [35].

## 4. Liposomal Encapsulation of Bioactive Compounds and Application to Food Matrices

Owing to their amphiphilic nature, liposomes offer a versatile platform for encapsulating both hydrophilic and hydrophobic food bioactive compounds. This dual-loading capacity facilitates the functional delivery of a wide range of nutraceuticals and functional ingredients. Various classes of food bioactive compounds, including vitamins, polyphenols, carotenoids, and omega-3 fatty acids, have been successfully encapsulated into liposomes. The application of liposome-encapsulated bioactives in food matrices is aimed at amplifying the antimicrobial potential, bioavailability, and bioaccessibility, increasing intestinal absorption, improving flavor profiles, and enhancing the overall functionality and quality of foods.

When liposomes are introduced into real food matrices, naturally occurring components, such as mineral ions, fats, proteins, and sugars, could decrease the physical stability of liposomes. For example, salt and sugars can raise the osmotic pressure gradient across the liposomal membrane, resulting in dehydration, disrupting electrostatic and hydrophobic interactions essential for membrane integrity. The pH of the target is another critical factor; for instance, under acidic conditions lipid ionization and hydrolysis may occur, leading to vesicle fusion, increased particle size, and loss of encapsulated compounds. At temperatures above the phase transition point of phospholipids, the bilayer loses much of its tightly packed structure. Consequently, processing and storage temperatures of foods become crucial for liposome stability, as elevated temperatures can also promote lipid oxidation of phospholipids due to their unsaturated fatty acid contents.

### 4.1. Vitamins

Fat-soluble vitamins (A, D, E and K) can be embedded within the hydrophobic region of the liposomal membrane, protecting them from oxidative degradation and improving their transfer into aqueous food matrices. Fan et al. [37] encapsulated fat-soluble vitamins in egg yolk phosphatidylcholine liposomes, and reported preferential localization within the hydrophobic bilayer. Their relative occupancy in the bilayer followed the order vitamin K > vitamin D > vitamin E > vitamin A. Over three months of storage, the vitamins showed a progressive migration toward the lipid–water interface. Increasing vitamin loading reduced bilayer fluidity, decreasing alkyl chain mobility and limiting water permeability. As oxidative reactions are promoted by the presence of water within the bilayer, this reduction in water flow correspondingly lowered the rate of oxidation. Furthermore, increased membrane rigidity restricted vitamin mobility, thereby improving physicochemical stability during storage [37].

Liu et al. [38] fortified mandarin juices with uncoated and alginate–chitosan-coated liposomal vitamin C. Surface coating by polymers protected the liposome core structure from oxidation and hydrolysis, and reduced the release of vitamin C after 90 days of storage at 4 °C. Although, aside from sensory analysis, pasteurization treatment and all assays were not performed in real food matrices, Marsanasco et al. [39,40] reported that soy phosphatidylcholine (SPC) liposomal vitamins could be a viable alternative to fortify beverages when their membranes were stabilized by stearic acid (SA) or calcium stearate (CaS). In both studies, food simulants, acetic acid solution, and distilled water were used to mimic aqueous foods, such as orange juice (pH < 4.6) and chocolate milk (pH > 5), in order to avoid the interference from other food constituents. The addition of SA or CaS as bilayer stabilizers reduced liposomal aggregation during storage, improved membrane packaging, and enhanced oxidative stability of plain SPC liposomes after pasteurization. Vitamin C, a thermolabile compound, also provided stability to liposomes against lipid oxidation. Pasteurized liposomes (vitamins C and E) were incorporated into commercial orange juice and chocolate milk for sensory evaluation, considering their recommended daily intake values. All liposomal vitamin-fortified orange juice samples were well accepted by panelists, whereas trained evaluators noted oxidized, metallic, and astringent flavors in SPC- and SPC:CaS-fortified milks compared to controls. No sensory differences were detected for SPC:SA-fortified milks.

Wechtersbach et al. [41] encapsulated ascorbic acid (AA) in DPPC and DPPC/cholesterol (DPPC/chol) liposomes, and evaluated their performance in fortified apple juice and fermented milk. The pH of liposomal AA was set to around 4, since leakage was more pronounced at pH 3 due to acid-catalyzed phospholipid hydrolysis. After 20 s pasteurization at 72 °C, only 10% of AA leaked from DPPC/chol liposomes, compared with over 60% leakage from DPPC liposomes under the same conditions. After 2 min of incubation, leakage increased to 33% and 86%, respectively. Apple juice, a complex medium containing trace amounts of redox active ions and partially oxidized phenols, is challenging for AA stability. When free AA was added to apple juice, over 90% degraded within 24 h at 25 °C. In contrast, more than 80% of AA remained in both types of liposome-fortified juices during the same period. In fermented milk matrix stored at 4 °C, free AA retained more than 50% of its initial content after 5 days of storage, but was fully degraded by day 26. Liposomal encapsulation tripled AA’s half-life in fortified milk, with 30% still remaining at the end of storage. The lower stabilization effect of AA by liposomes in fermented milk could be ascribed to its lipid components integrating into the membrane and increasing its permeability, or to enzymatic activity of bacterial or milk lipoprotein lipases that could hydrolyze DPPC.

### 4.2. Polyphenols

Polyphenols including resveratrol, quercetin, catechin, curcumin, and quercetin are well recognized for their health benefits as natural antioxidants; however, their poor solubility in water and limited bioavailability pose significant challenges for effective delivery [42]. Liposomal encapsulation offers a promising solution by enhancing the solubility of polyphenols and preventing their degradation during digestion. For example, liposomes shielded resveratrol from degradative factors such as light and oxidation, thereby increasing the amount of bioactive compound that reaches systemic circulation [43]. Similarly, encapsulation of quercetin and green tea catechins in liposomes increased their absorption rates in the small intestine and strengthened their antioxidant effects [44]. Studies show that polyphenols with higher lipophilic character can be more embedded in the liposome bilayer and thus yield higher encapsulation efficiency. Furthermore, liposomal encapsulation has been demonstrated to improve the in vitro bioaccessibility of propolis polyphenols such as caffeic acid phenethyl ester, galangin, pinobanksin, and pinocembrin, likely by providing protection to these phenolic compounds during simulated digestion [45].

Feng et al. [46] fortified orange juice (pH = 4) with uncoated and low-methoxy-pectin (LMP)-coated liposomes loaded with resveratrol and epigallocatechin gallate. After pasteurization (65 °C for 20 min), higher lipid oxidation occurred in fruit juices fortified with uncoated liposomes. On the other hand, LMP-coated vesicles likely formed a network-like gel due to the Ca^2+^ ions existing in orange juice. This gel structure was reversible after heating, contributing to increased stability during the subsequent storage. After 20 days of storage at 4 °C, all liposomes aggregated and released 8.92 to 15.26% of their encapsulated compounds, with coated liposomes exhibiting lower leakage.

Polyphenols are known to bind noncovalently to proteins, leading to an irreversible precipitation of protein–polyphenol complexes. Doum fruit extract encapsulated in liposomes was added to milk prior to fermentation [47] to prevent the interaction of polyphenols and milk proteins, therefore retaining antioxidant activity in yogurt samples. Higher concentration of liposomes had an initial negative effect on yogurt texture, though this impact diminished during storage. Similarly, adding olive leaf extract-loaded liposomes to set-type yogurt after fermentation of milk [48] masked its bitter and pungent taste, while lecithin in the formulation adsorbed water, reduced syneresis, and improved mouthfeel.

Altin et al. [49] fortified ayran (drinking yogurt) with cocoa hull waste phenolics encapsulated in chitosan-coated liposomes produced by high-pressure homogenization, and assessed the in vitro bioaccessibility of phenolics in fortified ayran samples over 15 days of storage. The liposomes added in spray-dried form in the presence of maltodextrin had better stability for phenolics compared to those added in liquid form (without maltodextrin), whereas liquid liposomes performed similarly to free phenolics.

Liquid and spray-dried liposomes loaded with nettle extract have been utilized as natural additives in cake to enhance functional value and extend shelf life. Whether added in liquid or dried form, liposomal encapsulation effectively masked the astringent taste of extract in fortified cakes. Only the cakes fortified with spray-dried liposomes showed no significant differences in yeast and mold counts compared to those containing potassium sorbate during 28-day storage. By comparison, cakes fortified with liquid liposome suspensions did not demonstrate similar protection, indicating that bare liposomes were insufficient to protect antimicrobial activity of extract under baking conditions, specifically at baking temperatures of approximately 220 °C and crumb temperature near 98 °C [50].

The antifungal properties of garlic extract-loaded phosphatidylcholine-oleic acid liposomes in wheat bread storage was evaluated by Pinilla et al. [51]. Both free garlic extract and liposome-encapsulated extract extended the mold-free period by at least 5 days compared to the control. After 11 days of storage, control bread slices were completely covered with mold, whereas at day 15, only four slices with free extract and two slices with liposomal extract were fully moldy, reflecting an increase in antifungal protection.

### 4.3. Carotenoids

Carotenoid pigments have strong antioxidant properties; however, their lipophilic nature presents challenges for incorporation into aqueous food systems and limits their bioavailability. Liposomal encapsulation is an effective strategy to increase both the stability and solubility of carotenoids such as lycopene and β-carotene [52]. By embedding these compounds within the hydrophobic bilayers of the liposome, they can be protected from oxidative degradation reactions. β-carotene, in particular, is highly susceptible to oxygen and light, and its encapsulation within liposomes has been shown to significantly extend its shelf life [53]. In a study, co-encapsulation of β-carotene and vitamin C together into liposomes provided synergistic protection against β-carotene degradation during storage [54].

Dutta et al. [55] utilized β-cryptoxanthin (β-crx)-loaded nanoliposomes in milk as a fortification strategy to improve dietary vitamin A intake. The β-crx molecules occupied intermolecular spaces within the bilayer, providing protection against oxidation during storage. Retention of β-crx in fortified milk exceeded 92% following three different heat-treatment regimes. Simulated gastrointestinal digestion indicated that β-crx fortification could contribute 17–48.32% of the daily-recommended vitamin A intake. Although this study did not evaluate lipid oxidation or perform sensory analysis in heat-treated fortified milks, the findings support liposomal delivery as a promising approach to address vitamin A deficiency through milk fortification.

### 4.4. Omega-3 Fatty Acids (DHA and EPA)

Omega-3 fatty acids, particularly docosahexaenoic acid (DHA) and eicosapentaenoic acid (EPA), are essential nutrients well known for their cardiovascular health benefits. In their free form, however, they are highly susceptible to oxidative degradation. Liposomal encapsulation offered an effective strategy to protect omega-3-rich fish oils from oxidative deterioration. The peroxide value of fish oil encapsulated in nanoliposomes increased at a slower rate compared to that of free oil, indicating improved oxidative stability. Sensory evaluation studies further reveal that the yogurt fortified with liposomal fish oil received consumer acceptance scores comparable to those of control yogurts without added fish oil [56].

Ojagh and Hasani [4] reported that nanoliposomal encapsulation reduced the oxidation of fish oil during 25 days storage at 4 °C. Breads fortified with liposomal fish oil showed higher loaf volume and lower hardness values than both the control and free fish-oil-fortified breads. The improvements were attributed to the interaction of lecithin with amylose and amylopectin, which helps prevent crumb hardening. Additionally, lecithin used in the formation of liposomes and glycerol employed in the hydration medium acted as an emulsifier, modifying the textural properties of fortified breads.

### 4.5. Other Bioactive Components

In recent years, liposomes have also demonstrated potential for the encapsulation of peptides [57], coenzyme Q10 (CoQ10) [58], probiotics [59], minerals [60], amino acids [61], and essential oils [62]. For instance, encapsulating probiotics in phospholipid vesicles shields them from harsh conditions (light, oxygen, gastric acid, bile), greatly improving their survival during digestion and promoting targeted release in the intestines [59]. Similarly, liposomal delivery of CoQ10 has proven effective. Niu et al. [58] found that a high-protein beverage fortified with liposomal CoQ10 achieved ~2.8-fold higher in vivo bioavailability of CoQ10 compared to non-encapsulated CoQ10. Liposomes have also been used to encapsulate protein peptides (and by extension amino acids), improving their stability and functionality in food matrices. Huang et al. [63] encapsulated bioactive peptides into liposomes modified with fatty acids of different chain lengths, thereby improving membrane compactness, hydrophobicity, and storage stability while enabling delayed peptide release during simulated digestion. For essential oils, *Cuminum cyminum* essential oil-loaded nanoliposomes incorporated into a chitosan coating reduced microbial growth and lipid oxidation in sardine fillets during 16 days of refrigerated storage [64].

In the dairy industry, liposomal encapsulation can also be employed for the targeted delivery of enzymes and antimicrobial peptides, such as bacteriosins. Nisin, a potent antimicrobial peptide, is widely used to extend the shelf life of cheese. However, when added directly to cheese milk in free form, nisin interacts with fats and proteins, reducing its availability for bacteria. Encapsulation in liposomes can prevent these interactions with food components, minimize interference with starter cultures during fermentation, and provide long-term preservation during ripening and storage. However, nisin can also interact with phosphatidyl membranes and induce leaking. This effect can be mitigated by modifying the lipid composition, e.g., using high-melting-point phospholipids and altering membrane properties. In a Cheddar cheese fermentation study by Laridi et al. [65], the stability of nisin-loaded liposomes was strongly influenced by the composition of the external environment. Liposomes were the least stable in whey, likely due to the higher concentration of bivalent ions (e.g., calcium and magnesium), with calcium identified as the most significant destabilizing factor for lipid membranes. The amount of nisin released also increased with higher milk fat content, possibly due to interactions between liposome and fat globule membranes that promote localization within the fat phase and destabilization of the liposomal bilayer.

Liposomal encapsulation can also be applied to enzyme delivery in cheese manufacture. Encapsulated enzyme cocktails, such as flavourzyme, neutral bacterial protease, and fungal protease and lipase, can accelerate cheese proteolysis and lipolysis, producing cheeses with a more mature texture and greater flavor intensity in a shorter ripening period. In contrast, free enzymes are prone to premature attack on substrate, which, in turn, results in excessive lipolysis and undesirable flavor and texture. During cheese fermentation, liposome-encapsulated enzymes, e.g., proteases/peptidases, concentrate predominantly in the curd, whereas free enzymes are dispersed throughout the whole-milk matrix, resulting in low retention of flavor-developing enzymes in the curd. Nonetheless, encapsulation of peptides and enzymes in liposomes can be limited by low encapsulation efficiency and protein instability under liposome preparation conditions [66,67,68].

Liposomal technology has also shown promise for mineral fortification in dairy systems. For instance, milk fortified with ferrous sulfate-loaded liposomes [69], prepared by optimizing Tween 80, cholesterol, ferrous sulfate concentration, sonication strength, and hydrating medium, remained stable after heat sterilization (100 °C, 30 min), and for one week of storage at 4 °C. Fortified milk containing 15 mg/L iron showed no precipitation, coagulation, or perceptible changes in color and off-flavor compared with control milk. Beyond stability improvements, liposomal encapsulation can significantly enhance mineral bioavailability. In a recent human trial, an iron-fortified liposomal supplement achieved more sustained and elevated iron uptake than a conventional supplement, with similar absorption benefits observed for magnesium and calcium [70].

## 5. Limitations of Liposomes

Although liposomal encapsulation, particularly for food and oral delivery systems, is considered a promising system so far, it has been limited by several weaknesses. These include low physical stability, sensitivity to environmental stresses encountered during food production (such as fluctuations in pH, ionic strength, and temperature), a tendency for aggregation or fusion, and rapid leakage of encapsulated compounds over storage time [30]. Moreover, gastric acid, bile salts, and pancreatic lipases in the gastrointestinal tract have also detrimental effects on liposomal membrane integrity, which lead to premature release and degradation of encapsulated bioactives [45,71]. Consequently, liposomes alone may not provide sufficient protection for sensitive bioactives during processing, storage, or gastrointestinal passage.

To overcome these limitations, coated liposomes have been developed by depositing biopolymers—primarily proteins and polysaccharides—on the liposomal surfaces through electrostatic interactions. This surface-coating strategy enhances the structural integrity, stability, encapsulation efficiency, and functional performance of the liposomes under varying physicochemical conditions. Common food-grade coating materials include chitosan [11,57], pectin [72,73], alginate [10], apple fiber [74], and whey proteins [75], each offering different advantages depending on the target application. Multilayer coatings with oppositely charged biopolymers can further strengthen liposomal membranes and modulate release kinetics, particularly for gastrointestinal or complex food matrices [38,76]. For example, fiber-coated liposomes have demonstrated enhanced sustained caffeine release and provided higher stability in stomach acid, as well as gastro intestinal fluids [74]. The choice of coating material is guided by considerations of biocompatibility, negligible toxicity, and neutral sensory characteristics. Depending on polymer type and molecular weight, coatings can form a physical barrier which hinders destabilization, enzymatic attack, and degradation reactions. Surface coating may also enhance mucoadhesive properties of liposomes; for instance, chitosan-coated liposomes exhibited improved mucoadhesiveness and delayed release profiles, making them suitable for oral or mucosal delivery systems [57,77].

Another promising approach to overcoming the intrinsic limitations of conventional liposomes is their incorporation into hybrid delivery systems, in which liposomes are embedded within solid or semi-solid matrices such as hydrogels, electrospun nanofibers, particles, emulsions, or edible films. These hybrid architectures provide a supportive framework that improves the physical and chemical stability of liposomes, prevents premature leakage, and facilitates a more controlled release of the encapsulated bioactives under physiological or food processing conditions. For example, liposome-loaded nanofibers offer a dry, high-surface-area platform that maintains vesicle integrity, while a hydrogel-based system ensures sustained release and mucoadhesion in oral delivery applications [16,18,78]. Moreover, hybridization provides an additional barrier for liposomes against harsh gastrointestinal environments and reduces their interactions with food matrix components, thereby maintaining bioactive potency and enhancing bioavailability. Consequently, the integration of liposomes into multifunctional hybrid systems has emerged as a robust approach to broaden their applicability in functional food, nutraceutical, and edible biomedical formulations.

## 6. Liposome-Loaded Hybrid Delivery Systems

**Figure 3:**
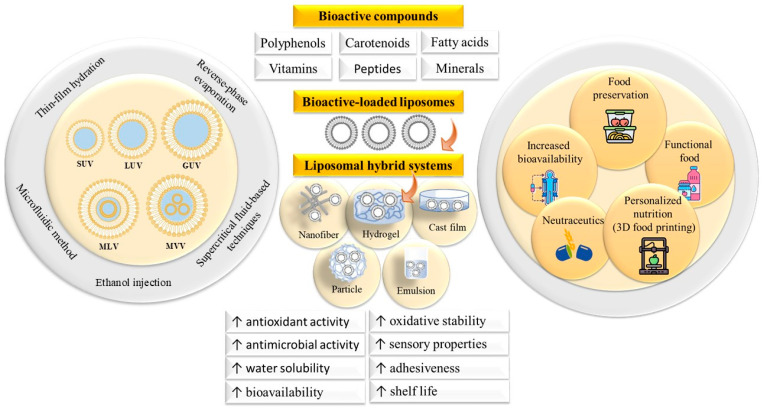
Illustrative summary of the liposomal hybrid systems for food applications. SUV: small unilamellar vesicle, LUV: large unilamellar vesicle, MLV: multilamellar vesicle, MVV: multivesicular vesicle, and GUV: giant unilamellar vesicle.

Liposome-loaded hybrid delivery systems have rapidly emerged as versatile platforms that enhance the stability, bioavailability, and functional efficacy of sensitive bioactives in food-related applications. This section explores current trends, innovations, and challenges in the development and use of liposome-loaded hybrids, including nanofibers, cast films, hydrogels, particles, and emulsions. By integrating liposomes with various polymeric and colloidal matrices, these systems overcome many limitations of conventional liposomes, particularly regarding physical stability, controlled release, and targeted delivery, thereby unlocking novel possibilities in active packaging, oral delivery, and taste and odor masking, and showing promising potential in emerging technologies such as 3D food printing (Figure 3).

To provide a consolidated perspective, Table 2 provides a comparative overview of representative liposome-based hybrid food delivery systems, summarizing their encapsulated compounds, key functional improvements, application areas, advantages, and limitations as reported in recent literature. The following subsections synthesize key findings that highlight the potential of these hybrid structures in food-related applications.

### 6.1. Liposome-Loaded Nanofibers

Integrating liposomes into electrospun nanofiber matrices addresses inherent challenges of conventional liposomes, such as limited stability, aggregation, and leakage, while leveraging the advantages of nanofibers, including high surface area, tunable porosity, and controllable release profiles. Beyond food packaging and preservation, liposome-loaded nanofiber hybrids hold great promise for next-generation functional foods designed to improve nutritional value, mucoadhesiveness, bioavailability, and targeted delivery. Incorporating stimuli-responsive features triggered by pH, enzymes, or temperature could enable precise site-specific release, while tailoring polymer–liposome interactions may boost performance for various bioactives. This section provides an in-depth overview of recent progress in liposome-loaded nanofiber systems, focusing on their design, functional properties, and application potential in food-related uses.

Across a variety of nanofiber matrices, including pectin, pullulan, gelatin, zein, polyethylene oxide (PEO), and chitosan, consistent findings demonstrate enhanced bioaccessibility, stability, and sustained release kinetics of encapsulated bioactives (Table 3). These bioactives range from hydrophilic compounds like anthocyanins, green tea extract, and peptides, to hydrophobic agents including propolis, curcumin, β-carotene, and essential oils, with these hybrid systems often outperforming single-carrier counterparts in both protection and release control. Moreover, these hybrids exhibit improved mechanical strength, mucoadhesiveness, and UV barrier properties, making them highly suitable for applications like oral delivery and active packaging.

For example, to improve the stability and intestinal absorption of anthocyanins, a class of polyphenols with inherently low bioavailability due to rapid degradation during intestinal digestion, Kalıntas Caglar et al. [17] developed black mulberry extract (BME)-loaded liposomal pullulan/pectin nanofibers. Compared to nanofibers loaded with free BME, these liposomal nanofibers demonstrated enhanced cellular release of four major anthocyanins in Caco-2 models, indicating improved transepithelial transport, a key step toward higher in vivo bioavailability. Furthermore, liposomal incorporation significantly increased mucoadhesiveness, potentially prolonging gastrointestinal residence time, and nearly doubled anthocyanin bioaccessibility. Similarly, Feng et al. [93] addressed the challenge of delivering therapeutic peptides and proteins to the colon by producing a multi-unit liposomal nanofiber mat via coaxial electrospinning. The fiber core consisted of pectin-coated salmon calcitonin (sCT)-loaded liposomes, while the shell was composed of sodium alginate. This core–shell structure provided sustained, colon-specific release (65.2% in simulated colon fluid versus 47.8% from uniaxial liposomal fibers), with the pectin coating enhancing resistance to enzymatic and acidic degradation and the alginate shell acting as a pH-responsive barrier to prevent premature peptide leakage. Consequently, burst release in gastric and intestinal simulations was minimized, retaining 88% peptide bioactivity.

Building on liposomes’ ability to encapsulate hydrophobic compounds alongside the mucoadhesive properties of gelatin and zein polymers, Karakas et al. [16] developed multifunctional, edible wound-healing nanofiber dressings loaded with liposomal propolis via coaxial electrospinning. Designed to target oral mucosal injuries, where a moist and bioactive-rich environment is critical for effective tissue repair, the system protected propolis flavonoids from degradation and enabled a gradual pH-dependent release at the wound site to extend antimicrobial and anti-inflammatory effects and cell proliferative effects. This entirely food-compatible platform holds strong promise for safe oral healthcare, particularly for minor oral wounds or irritations, and addresses the stability and delivery challenges while aligning with emerging interest in edible biomedical materials as alternatives to synthetic wound dressings.

Another hydrophobic polyphenol, curcumin, with antioxidant and antimicrobial properties, was encapsulated within liposome-embedded electrospun gelatin and zein by Alehosseini et al. [79]. Maintaining liposomal integrity within the nanofiber matrix is essential to protect curcumin from oxidation and photodegradation until release. Polymer choice and co-encapsulation strategies can be tailored to align the polarity and moisture content of the target food matrix. For instance, zein-based mats offered superior protection during storage and enabled more controlled release in hydrophobic food simulants, whereas gelatin-based mats released curcumin more rapidly and exhibited lower thermal stability. Co-encapsulation of curcumin with green tea extract improved the stability of gelatin fibers but had little impact on zein fibers [79].

**Table 3:** Overview of liposome-loaded nanofiber delivery systems in food applications.

Nanofiber Matrix Components	Ingredients and Size of Liposomes	Production Method of Liposome	Liposome-Encapsulated Bioactive	Results	Ref
Pectin/Pullulan	Soybean lecithin; 76 nm	Thin-film hydration + ultrasonication	Black mulberry extract (BME)	Uniform distribution of liposomes in the defect-free fiber structure; The higher cellular (Caco-2) release of anthocyanins in liposomal-BME loaded nanofiber	[17]
Zein/Gelatin	Soybean lecithin, polyoxyethylene sorbitan monooleate (Tween 80); 370 nm	Thin-film hydration + ultrasonication	Propolis	Enhanced mucoadhesiveness; Edible oral wound-healing material; Improved healing of oral wound and proliferation	[16]
	Soybean lecithin; 154 nm	Ethanol injection	Curcumin	Gelatin coatings preserved fatty food simulants; Zein-based coatings preserved moisture of foods	[79]
Polyethyleneoxide (PEO)	Soybean lecithin, cholesterol, stearylamine; 123–165 nm	Thin-film hydration	Basil essential oil	Maintaining the quality of chilled pork during 4 days of storage	[94]
Alginate/PEO-Polyvinyl alcohol (PVA), Pectin	Soybean lecithin, cholesterol; 26–32 nm	Thin-film hydration	Salmon calcitonin	Enhanced bioavailability (colon-targeted delivery)	[93]
Chitosan	Soybean lecithin, cholesterol; 144 nm	Thin-film hydration	Tea tree oil	Protection against *Salmonella* spp. in chicken and extended shelf life	[95]
PEO	Soybean lecithin, cholesterol; 315 nm	Thin-film hydration	SiO_2_-eugenol nanoparticles	Stable antioxidant activity during 60-day storage of beef	[96]
PEO/PVA	Hydrogenated soybean lecithin; 194 nm	Ethanol injection	β-carotene	Improved photostability	[97]

To further enhance photostability and water dispersibility of hydrophobic bioactives for aqueous food applications, de Freitas Zômpero et al. [97] incorporated β-carotene-loaded nanoliposomes into electrospun ultrathin fibers of PVA and PEO. The addition of nanoliposomes significantly altered the feed solution rheology, particularly with PVA, indicating strong interactions with the hydrophilic polymer chains. Importantly, liposomal structures remained intact upon release from the fiber matrix, physically protecting β-carotene from light penetration and oxidative damage.

Given their controlled and stimuli-responsive release capabilities, liposome-loaded nanofibers also show significant promise in active packaging of perishable foods. For example, Li et al. [94] incorporated cationic liposomes encapsulating basil essential oil (BEO) in PEO electrospun nanofibers. These fibers exhibited antibacterial activity against *Listeria monocytogenes*, which secretes a broad-range phospholipase-C capable of hydrolyzing phosphatidylcholine. This pathogen-specific enzyme triggered accelerated BEO release from the liposomes, minimizing unnecessary loss of active compounds and effectively maintaining pork quality during chilled storage for up to 4 days. Similarly, Cui et al. [95] developed chitosan-based electrospun nanofiber liposomes loaded with tea tree oil (TTO) to deliver natural antimicrobials directly to fresh meat surfaces. This approach maintains meat texture, color, and flavor, which are key to consumer acceptance. Encapsulation of TTO in liposomes reduced volatility and protected bioactivity, enabling controlled, sustained antimicrobial release. When applied to chicken meat, the system achieved approximately a 5-log reduction in *Salmonella* over 4 days of storage at both 12 °C and 25 °C without sensory deteriorations. Extending these strategies, Cui et al. [96] fabricated a multi-barrier protection system that combines inorganic nanoparticle adsorption, liposomal encapsulation, and nanofiber immobilization. Eugenol-adsorbed silica (SiO_2_) nanoparticles encapsulated in liposomes were immobilized within electrospun PEO nanofibers, providing sustained antioxidant activity in beef for up to 60 days. Silica enhanced the physical stability of the liposomes, reduced eugenol volatility, and provided a rigid support that limited lipid bilayer deformation.

Further research should emphasize personalized nutrition and nutraceutical delivery, tailoring these systems to individual dietary needs and health conditions. Most findings remain limited to in vitro or model systems, highlighting the need for validation in real food matrices and in vivo conditions for industrial translation.

### 6.2. Liposome-Loaded Cast Films

Effective food preservation requires balancing the release of active compounds from edible films, as rapid release is undesirable for long-term preservation, while targeted, accelerated release during mid-storage can inhibit pathogenic bacterial growth. Embedding liposomes in cast films addresses this challenge by enabling controlled bioactive delivery, enhancing barrier and optical properties, and offering biodegradability, thereby providing a food-grade, sustainable alternative to conventional synthetic packaging. Adding stimuli-responsive release and multi-bioactive loading could further strengthen their commercial potential.

Liposomes encapsulating various antimicrobial and antioxidants including essential oil [81,82], inorganic nanoparticles [81], phenolic extracts [80,98,99], saffron carotenoids [83], and bioactive peptides [100] have been incorporated into edible cast films composed of different polymers such as chitosan, gelatin and pullulan, sodium alginate (SA), carboxymethyl cellulose (CMC), konjac glucomannan (KGM), polyvinyl alcohol (PVA), and sodium caseinate (NaCas). These liposome-loaded cast films serve as biodegradable food coatings that enable sustained, controlled release or encapsulated compounds while enhancing key film properties including water vapor and oxygen permeability, thermal stability, mechanical strength, and UV transmittance (Table 4).

For example, chitosan films loaded with liposomes encapsulating both thyme essential oil and Cu nanoparticles demonstrated a sustained, controlled release of 65.17% of essential oil and 15.17% of nanoparticles over seven days of litchi fruit storage [81], which resulted in delayed pericarp browning and reduced microbial growth. Similarly, gelatin–chitosan biopolymer films loaded with nanoliposome-encapsulated *Nicotiana tabacum* (tobacco) leaf extract (TE) exhibited significant antimicrobial activity against *Salmonella enterica* and *Pseudomonas aerogenosa*, alongside lower water vapor permeability and UV transmittance, as well as enhanced thermal stability and mechanical strength [98]. On the other hand, gelatin/chitosan films loaded with liposome-encapsulated betel leaf extract (L/BLEE) exhibited lower antioxidant activity compared to films loaded with free BLEE-loaded films, likely due to stronger entrapment within the film matrix that slowed the diffusion of active phenolic compounds, leading to a more sustained but less immediate antioxidant effect [99]. In a similar manner, Najafi et al. [83] incorporated antioxidant saffron extract-loaded nanoliposomes in pullulan film, resulting in films with appealing flavor and color, as well as increased oxygen barrier properties. However, in both studies, liposome loadings depending on the inclusion ratio compromised mechanical integrity of the polysaccharide films, indicating possible phase separation or incompatibility during drying, which highlights the need for formulation optimization when integrating liposome-based systems into cast films. On the other hand, when Montero et al. [100] incorporated nanoliposomes encapsulating a bioactive shrimp peptide fraction into NaCas protein films, the liposomes acted as plasticizing agents within the protein network, enhancing chain mobility without compromising film integrity.

Tailoring liposome surfaces to develop intelligent packaging systems capable of responding to changes in the storage environment is particularly advantageous in food packaging. This approach helps to minimize premature loss of bioactives and ensure precise, timely release of antimicrobial or antioxidant agents exactly when and where they are needed. To this end, Liang et al. [82] engineered pH-responsive liposomes encapsulating Alpinia galanga essential oil, which were immobilized within a PVA/KGM network. The liposome surfaces were initially modified with hexadecyl trimethyl ammonium bromide (CTAB) to impart positive charge, followed by coating with negatively charged γ-Polyglutamic acid (γ-PGA). The pH-responsive behavior of γ-PGA enabled a triggered release of the essential oil from PVA:KGM composite films in response to pH decreases induced by microbial activity in the citrus fruit-packaging environment. Similarly, Feng et al. [80] developed SA/CMC edible films loaded with pH-responsive liposomes containing green tea extract to coat blueberries. Compared to controls, the liposome-loaded cast film effectively reduced water loss and preserved texture by slowing the decline in hardness, and maintained a more consistent weight loss rate over two weeks of storage. The pH-responsive liposomes likely facilitated targeted release of antioxidant green tea polyphenols in response to metabolic changes in the fruit microenvironment, thereby delaying senescence.

### 6.3. Liposome-Loaded Hydrogels

Liposomes and hydrogels have long been recognized as versatile delivery platforms, each offering distinct functional advantages. Increasingly, research has focused on combining these systems into liposome–hydrogel hybrids, which unite the encapsulation and controlled release capabilities of liposomes with the structural integrity and tunable physicochemical properties of hydrogels [101].

A defining feature of liposome–hydrogel hybrids is their two-stage release mechanism: liposomes are first liberated from the hydrogel matrix, followed by the gradual release of the encapsulated compounds [102]. Incorporating liposomes into hydrogel networks provides several added benefits, including improved solubility of hydrophobic compounds in aqueous systems, enhanced stability of labile bioactives, sustained and stimuli-responsive release, increased mucoadhesiveness, and more efficient targeted delivery [18,88,103]. Despite these promising features, current research has primarily focused on pharmaceutical and biomedical applications, with limited exploration in food applications [88]. Recent studies of liposome-loaded hydrogels in food-related applications are summarized in Table 5.

To encapsulate hydrophobic propolis extract within hydrogels composed of xanthan gum and salep polymers, Saroglu et al. [104] developed a liposomal gel system. In their initial study, they examined the formulation-dependent structural changes occurring in the hydrogel network upon incorporation of liposomes. The liposomal vesicles preserved their integrity within the bird-net porous hydrogel structure. Moreover, incorporating liposomes enhanced the structural integrity and rheological properties of the xanthan gum/salep hydrogels, with higher salep ratios further improving the gel strength [104]. Building on these findings, Saroglu et al. [18] conducted a follow-up study using these hydrogels to deliver liposomal propolis. They demonstrated that choice of surfactant (AMP or Tween 80) employed to increase propolis loading significantly influenced gel rheology, as well as the gastrointestinal stability and bioaccessibility of individual propolis phenolics. Notably, AMP-based liposomal gels showed superior performance. Due to their entrapment in the hydrogel matrix, a substantial number of vesicles remained intact after gastric digestion and swelled during the intestinal phase. The resulting lysophospholipids could form micelles with bile salts, supporting efficient nutrient delivery. A comparable effect related to improved gel structure and its impact on release and bioaccessibility of encapsulated compound was also observed by Liu et al. [88], who enhanced gastric protection of quercetin by embedding liposomes into a chitosan–gelatin matrix and tailoring the hardness of liposomal gels. Hard lipogels exhibited more significant physical breakdown, whereas soft lipogels degraded faster and released higher amounts of liposomes and quercetin. Likewise, Mousavi et al. [86] found that increasing the hydrogel-to-liposome ratio resulted in a denser polymer network, which slowed down curcumin release from liposome-loaded *Alyssum homocarpum* seed gum and alginate gels cross-linked with CaCl_2_ during simulated digestion and storage. Higher temperatures caused phase changes in the lipid bilayer and accelerated phospholipid breakdown, and, notably, higher hydrogel-to-liposome ratios delayed curcumin release. Moreover, Pasban et al. [105] reported similar structure-dependent behavior in denser SPI/alginate liposomal gels, yielding low phenolic release over 28 days of refrigerated storage, likely due to restricted water mobility. These gels exhibited higher phenolic release during acidic gastric stage but lower release and degradation in the intestinal digestion phase. Interestingly, Liu et al. [87] exploited the vesicle-forming ability of conjugated linoleic acids (CLAs) similar to lecithin to fabricate a hydrogel-liposome structure using CLA and chitosan (CS). Curcumin-loaded oligo-CLA nanovesicles (OCLAVs) were incorporated into β-glycerophosphate (β-GP)-CS gel matrix. These vesicle-based hybrid gels displayed a compact, shrunk-pore structure that effectively delayed curcumin release (51.23%) compared to either the CS gel (93.37%) or OCLAVs alone, and retained their porous structure even after in vitro digestion.

Liposomal inks have been used in the fabrication of implantable patches and injectable microbeads for localized therapeutic delivery [106,107]. Translating these approaches to food systems, liposomal hydrogels can serve as printable bio-inks to encapsulate and protect sensitive food bioactives. By tailoring the chosen polymers and liposome surfaces to specific triggers such as pH, temperature, or enzymes, and adjusting the textural strength, these advanced hybrid gels—characterized by higher water content, tunable texture and mucoadhesiveness, and the ability to carry both hydrophilic and hydrophobic compounds—offer significant potential for next-generation 3D functional foods. In particular, they may provide promising solutions for personalized nutrition, including formulations designed for individuals with dysphagia or swallowing difficulties, who may be at risk of dehydration and insufficient intake of essential minerals, and vitamins.

### 6.4. Liposome-Loaded Particles

Biopolymer particle matrices not only protect liposomal structures during drying, storage, and digestion, but also enable the conversion of aqueous liposomal suspensions into solid forms. Those liposomal powders, which can be easily regenerated upon aqueous dispersion, have great potential as ingredients for a wide range of functional foods. This section highlights key examples of liposome-loaded particle systems and their roles in advancing food-grade delivery technologies. Recent studies on liposome-loaded particles related to food applications are summarized in Table 6.

Liposomes encapsulating various bioactives, including phenolic extracts [109], protein hydrolysates [84], and anthocyanins [9], have been stabilized by using carriers such as maltodextrin, alginate, and trehalose through spray- and freeze-drying. When alginate was evaluated as a carrier for liposomal suspensions [108], it effectively preserved the size and shape of liposomes during drying and rehydration, while proving stability across different pH conditions. The reconstituted liposomes exhibited low viscosity compatible with beverage fortification. In another study, trehalose was successfully utilized as a carrier during spray-drying of liposomes loaded with *Tilapia viscera* hydrolysate [84]. These dried liposomes retained their antioxidant and ACE inhibitory activities even through simulated digestion.

Surface modification of liposomes played a crucial role in maintaining structural integrity and stability of encapsulated compounds during spray-drying and powder reconstitution. Liposomes coated with chitosan and whey protein, encapsulating *Rosa pimpinellifolia* fruit extracts [109] and anthocyanin-rich black carrot extract [9], were converted into dry powders by spray-drying using maltodextrin as a carrier. In both studies, coated liposomes retained their structure during spray-dying and demonstrated enhanced chemical stability after reconstitution. Although liposome powders exhibited three-fold higher in-vitro phenolic bioaccessibility, cellular uptake and transepithelial transport of phenolics showed no significant differences among formulations. Similarly, the ability of phenolics to suppress fatty acid-induced hepatocellular lipid accumulation was strongly dependent on the encapsulation matrix and method [109].

Hybrid liposome-based particles can also be used to mask the odor and taste of bioactive compounds used for food fortification. For example, Farahmand et al. [85] employed cinnamon essential oil (CEO)-loaded liposomes as the dispersed (core) phase, with a low-viscosity alginate solution serving as the continuous (shell) phase. Both solutions were pumped through a microfluidic channel to form the hybrid particles. Beverage samples fortified with hybrid liposomal CEO released about 20% less cinnamaldehyde in the headspace compared to liposomal CEO alone, demonstrating the effectiveness of the alginate shell in reducing aroma volatility and migration.

### 6.5. Liposome-Loaded Emulsions

An emulsion is a colloidal system in which one immiscible liquid is dispersed as small droplets throughout another, typically involving oil and water. In addition to their role as active carriers, liposomes have recently attracted attention as potential stabilizers in emulsions due to their ability to adsorb at the oil–water interface and form colloidal barriers in the absence of conventional surfactants [110]. Recent studies on liposome use in emulsions to enhance the physical and oxidative stability of bioactives are summarized in Table 7.

The close packing of emulsion droplets, coupled with a thicker liposomal layer surrounding them, can lead to increased structural consistency and reduced oil oxidation, both of which are crucial for emulsion-based food formulations that require structural and oxidative stability. This improved stability of liposome-containing emulsions was demonstrated by Pascual-Silva et al. [111] and Sun et al. [92]. In water-in-oil (W/O) emulsions stabilized by liposomal dispersions, the close packing of water droplets resulted in superior consistency compared to the control emulsions [111]. The thicker liposomal layer also likely reduced primary oxidation during homogenization and TBARS formation during cold storage. Sun et al. [92] showed that emulsions stabilized with freshly prepared liposomes or rehydrated lyophilized liposomes maintained stability across varying levels of pH, ionic strength, temperature, and storage duration, with acceptable oxidation stability. Furthermore, the pro-oxidant activity of iron in emulsions can also be mitigated by encapsulating iron within liposomes, isolating it from the emulsion interface [90]. This protection depended on the phospholipid-to-iron ratio: a high ratio reduced hydroperoxides and secondary oxidation products, while a low ratio promoted oxidative degradation, due to the localized iron accumulation and the generation of reactive intermediates in liposomal membranes. The protective effect of liposomes against heat degradation of encapsulated compounds in emulsion was demonstrated by Pattnaik and Mishra [91]. They incorporated liposome-loaded double emulsions encapsulating vitamins A, D, B_9_, and B_12_ into low-fat biscuits, achieving significantly improved vitamin retention during baking, with retention rates of 30.98–34% for vitamin B_12_, 24–32.63% for B_9_, 29.17–40% for vitamin A, and about 18% for vitamin D.

Emulsions can also be used for coating food surfaces. Al-Moghazy et al. [89] evaluated a coating emulsion combining liposomes encapsulating thyme essential oil (TEO) with chitosan to extend the shelf life of Karish cheese, a popular soft cheese in Egypt. Cheese samples coated with these liposomal TEO–chitosan emulsions maintained acceptable appearance and microbial quantity up to four weeks, while uncoated samples or those coated with chitosan solution alone exhibited visible microbial growth by the second week.

Unlike the other studies listed in Table 7, Trucillo et al. [112] employed the SuperLip (supercritical-assisted) process to entrap O/W emulsion directly within the liposomal aqueous core. This approach provided protection against degradation, as demonstrated by a smaller reduction in antioxidant activity. Liposomes loaded via this method exhibited submicron size distribution and enhanced stability, confirming that emulsion entrapment into the inner compartment of liposomes offers a more effective and protective environment for sensitive bioactives.

## 7. Conclusions and Future Perspectives

Liposome-based hybrid delivery systems represent a significant advancement in food and nutraceutical science, combining the encapsulation efficiency, unique amphiphilic structure, and controlled-release capabilities of liposomes with the structural, barrier, and functional advantages of solid and semi-solid matrices such as hydrogels, nanofibers, cast films, particles, and emulsions. These systems may address many of the limitations associated with conventional liposome formulations, including poor stability, leakage, and environmental sensitivity.

The studies summarized in this review confirm that these hybrid systems offer improved physicochemical stability, enhanced protection of sensitive bioactives during processing and storage, and more controlled release profiles, particularly under gastrointestinal conditions. The use of food-grade ingredients and scalable preparation methods further underlines the practical potential of these systems for the food industry. Moreover, the integration of smart functionalities, e.g., pH sensitivity, enzyme responsiveness, or targeted gastrointestinal delivery, into liposome-loaded platforms also holds great promise for the development of next-generation functional foods. Additionally, the potential of embedding liposomes in 3D-printed foods could open a new avenue for creating personalized functional foods tailored to individual nutritional needs, preferences, and health conditions.

Despite these advances, several challenges must be addressed before large-scale commercialization becomes feasible. Key barriers include ensuring long-term physicochemical stability, preventing lipid oxidation and undesirable interactions with other food ingredients, achieving reproducibility at industrial scale, and developing cost-effective, regulatory-compliant production methods. Additionally, limited knowledge exists on their performance in actual food matrices, under commercial processing, and across extended distribution and storage. Addressing these gaps will require systematic studies under realistic supply chain conditions and comprehensive safety and consumer acceptance evaluations.

Future research should focus on (i) the scalability and process optimization—translating laboratory formulations into robust, economically viable manufacturing protocols; (ii) advanced particle engineering—fine-tuning size, composition, and surface characteristics for precise, targeted release and flavor masking; (iii) integration with intelligent functionalities—such as pH, enzyme, or temperature sensitivity to enable responsive delivery; (iv) long-term performance evaluation—including stability, bioactive retention, and release kinetics during extended storage and distribution; and (v) application diversification—incorporating hybrid systems into emerging technologies, such as 3D-printed foods, for personalized nutrition.

With continued innovation and interdisciplinary collaboration, liposome-based hybrid systems have the potential to evolve from proof-of-concept formulations into market-ready solutions that enhance food quality, safety, and functionality, ultimately supporting the development of next-generation functional foods tailored to consumer needs.

## Figures and Tables

**Table 2:** Comparative functional performance of liposome-loaded hybrid systems across different matrices.

Delivery Matrix	Liposome-Encapsulated Compounds	Functional Improvements	Application Areas	Advantages	Limitations/Challenges	Ref.
Electrospun nanofibers	Anthocyanins; propolis; curcumin	Bioaccessibility; mucoadhesion; sustained release; antioxidant stability	Oral delivery; wound healing; active food packaging	Very high surface area; tunable release profiles; inherent mucoadhesiveness	Scale-up difficulties, low loading of active compound, limited variety of food-grade polymers	[16,17,79]
Edible films	Green tea extract; essential oils; saffron extract	Mechanical and barrier properties; shelf-life extension; aroma masking	Coatings of perishable fruits and meats; active packaging	Biodegradable; consumer and environment-friendly format	Potential odor migration; moisture sensitivity; limited loading capacity	[80,81,82,83]
Spray-dried particles	Protein hydrolysate; anthocyanins; essential oils	Stability; good rehydration properties; maintained bioactivity	Functional ingredient incorporation into dry foods	Extended shelf life; good rehydration	Thermal degradation risk of heat-labile bioactives	[9,84,85]
Hydrogels	Propolis; curcumin; quercetin	Structural integrity; mechanical strength; viscoelasticity; gastrointestinal stability; bioaccessibility	Oral delivery in food matrices; customized nutrition; edible food gels/sauces	High water content; tunable rheology	Limited mechanical stability under stress; syneresis and potential microbial growth (without preservatives) during storage	[18,86,87,88]
Emulsion	Essential oils: iron; vitamins; β-carotene	Oxidative and physical stability; bioaccessibility; protection during storage and thermal processing; reduced lipolysis	Dairy products; beverages; sauces; bakery applications	Especially suited for lipophilic bioactives; easy to scale up	Prone to phase separation over time; requires surfactant/emulsifier compatibility with food matrix	[89,90,91,92]

**Table 4:** Overview of liposome-loaded cast films in food applications.

Cast Film Matrix Components	Ingredients and Size of Liposomes	Production Method of Liposome	Liposome-Encapsulated Bioactive	Results	Ref
Chitosan	Soybean lecithin, cholesterol; 190 nm	Thin-film hydration	Copper nanoparticles, thyme essential oil	Better fresh-keeping effect on litchi	[81]
Bovine gelatin (B type)/Chitosan	Soybean lecithin, Tween 80; 110 nm	Thin-film hydration	*Nicotiana tabacum* (Tobacco) extract	Improved antibacterial efficacy against *Salmonella enterica* and *Pseudomonas aerogenosan*	[98]
Esterified konjac glucomannan (KGM)/Polyvinyl alcohol (PVA)	Soybean lecithin, cholesterol, hexadecyl trimethyl ammonium bromide (CTAB), γ-polyglutamic acid (γ-PGA, coating material); 100–300 nm	Thin-film hydration	Alpina galangal essential oil	pH-responsive liposomes endow films with intelligent controlled-release behavior; Extended the shelf life of citrus fruits	[82]
Sodium alginate/Carboxymethyl cellulose	Soybean lecithin, Tween 80; 810 nm	Thin-film hydration	Green tea extract	Improved mechanical strength and antioxidant capacity; Extended shelf life in blueberry packaging	[80]
Gelatin/Chitosan	Soybean lecithin, cholesterol; 195–450 nm	Thin-film hydration + ethanol injection	Betel leaf extract	Improved antioxidant activity and UV transmittance; Reduced visible light transmittance; Reduced release rate	[99]
Pullulan	Rapeseed lecithin; 119–138 nm	Thin-film hydration + ultrasonication	Saffron extract	Reduced oxygen permeability; Slightly weakened mechanical strength	[83]
Sodium caseinate	Soybean lecithin; 94–99 nm	Thin-film hydration+ ultrasonication	Shrimp peptide fraction	More water soluble and mucoadhesive films; Enhanced sensory properties	[100]

**Table 5:** Overview of liposome-loaded hydrogels in food applications.

Hydrogel Matrix Components	Ingredients and Size of Liposomes	Production Method of Liposome	Liposome-Encapsulated Bioactive	Results	Ref
Xanthan gum/Salep	Soybean lecithin (70% phosphatidylcholine, PC); 94.21 nm	Thin-film hydration	-	Liposome incorporation to gel enhanced viscoelastic behavior; gel hardness and structural stability	[104]
	Soybean lecithin (70% PC), Ammonium phosphatide (AMP), Tween 80; 80.97–179.30 nm	Thin-film hydration	Propolis extract	Liposome-loaded gels had higher mucoadhesion; AMP-based liposomal gels exhibited better structural and functional properties; The bioaccessibility of phenolics significantly increased compared to liposomes	[18]
Alginate/*Alyssum homocarpum* seed gum	Soybean lecithin, cholesterol; 0.06–0.13 mm (size of liposomal gel)	Ethanol injection	Curcumin	Improved thermal stability and curcumin bioaccessibility; Delayed release with increasing hydrogel ratio	[86]
Soy protein isolate (SPI)/Alginate	Soybean lecithin, cholesterol; 218.5–266.5 nm	Hydration + heating	Sumac extract	Ratio of SPI to alginate had a substantial impact on the release of phenolic compounds from liposomes; In intestinal conditions, denser matrices offered more protection and slower release for liposomes	[105]
Chitosan	OCLAVs (oligo-conjugated linoleic acid vesicles)	Thin-film hydration	Curcumin	Sustained release over 96 h (51.23% vs. 93.37% in control); Increased antioxidant activity up to 148.1%.	[87]
Chitosan/Gelatin	L-a-PC (Soybean > 94%)	Thin-film hydration	Quercetin	Liposome-loaded gels provided better gastric protection for quercetin (~40% less release) compared to free liposomes; Texture of gels influenced release behavior	[88]

**Table 6:** Overview of liposome-loaded particles in food applications.

Particle Matrix Components	Ingredients and Size of Liposomes	Production Method of Liposome	Liposome-Encapsulated Bioactive	Results	Ref
Alginate	Soybean lecithin, cholesterol; 252 nm	Ethanol injection	Cinnamon essential oil (CEO)	Masking the odor and taste of CEO in an acidic beverage	[85]
	Soybean lecithin; 98–612 nm	Thin-film hydration + ultrasonication	-	Enhanced stability of liposomes	[108]
Maltodextrin	Soybean lecithin, cholesterol, Tween 80, chitosan and whey protein (surface coating) uncoated: 86–95 nm; coated: 167 nm, 326 nm	Ethanol injection + high pressure homogenization	*Rosa pimpinellifolia* fruit extract (Rosa extract)	Protection of polyphenols against processing and digestive tract conditions	[109]
	Soybean lecithin, chitosan (surface coating); 82 nm	Thin-film hydration + ultrasonication	Black carrot anthocyanin extract (BCE)	Elevated physical and chemical stability of BCE	[9]
Trehalose	Soybean/rapeseed lecithin; 215–250 nm	Thin-film hydration + ultrasonication	Tilapia viscera hydrolysate	Enhanced structural and functional stability of liposomes	[84]

**Table 7:** Overview of liposome-loaded emulsions in food applications.

Emulsion Matrix Components	Ingredients and Size of Liposomes	Production Method of Liposome	Liposome-Encapsulated Bioactive	Results	Ref
W/O/W emulsion: Vegetable oil blend, milk protein isolate, trehalose	Soybean lecithin; 143–396 nm	Thin-film hydration + ultrasonication	Multivitamins (A, D, B_2_, B_9_, B_12_)	Vitamin A, D, B_12_ had >96% encapsulation; Improved stability and viscosity Freeze-dried liposomes enabled better vitamin retention	[91]
W/O emulsion: Sunflower oil	Phospholipids from shrimp waste; 143.13–190.5 nm	Thin-film hydration + ultrasonication	Antioxidant lipids	Enhanced oxidative and physical stability	[111]
O/W emulsion: Corn oil	Soybean lecithin, cholesterol, Tween 80; 91.3 3–339.5 nm	Thin-film hydration	Vitamin B_2_, Vitamin E, β-carotene	Improved stability; Higher vitamin B_2_ bioaccessibility; Smaller particle size	[92]
Chitosan, essential oil	Soybean lecithin; 42–175 μm	Reverse-phase evaporation	Thyme essential oil	Extended shelf life (up to 4 weeks); Reduced microbial growth; Stability of thymol and *p*-cymene	[89]
O/W emulsion: Rapeseed oil, Tween 20	Egg-yolk PC (~60%), cholesterol; 129–154 nm	Reverse phase evaporation + ultrasonication	Soluble iron (ferrous-Fe sulfate)	Free iron (Fe) and low PC:Fe ratio increased lipid oxidation	[90]
O/W emulsion: Isopropyl myristate, Tween 80	Egg-yolk PC (99%); 116–230 nm	Supercritical-assisted production	Lipophilic antioxidants (farnesol, limonene, linalool)	Higher encapsulation efficiency; Submicron particle size; Improved antioxidant stability	[112]

## Data Availability

Data will be made available on request.
